# Framework for Ranking Machine Learning Predictions of Limited, Multimodal, and Longitudinal Behavioral Passive Sensing Data: Combining User-Agnostic and Personalized Modeling

**DOI:** 10.2196/47805

**Published:** 2024-05-20

**Authors:** Tahsin Mullick, Sam Shaaban, Ana Radovic, Afsaneh Doryab

**Affiliations:** 1 Department of Systems and Information Engineering University of Virginia Charlottesville, VA United States; 2 NuRelm Pittsburgh, PA United States; 3 Department of Pediatrics University of Pittsburgh Pittsburgh, PA United States

**Keywords:** machine learning, AI, artificial intelligence, passive sensing, ranking framework, small health data set, ranking, algorithm, algorithms, sensor, multimodal, predict, prediction, agnostic, framework, validation, data set

## Abstract

**Background:**

Passive mobile sensing provides opportunities for measuring and monitoring health status in the wild and outside of clinics. However, longitudinal, multimodal mobile sensor data can be small, noisy, and incomplete. This makes processing, modeling, and prediction of these data challenging. The small size of the data set restricts it from being modeled using complex deep learning networks. The current state of the art (SOTA) tackles small sensor data sets following a singular modeling paradigm based on traditional machine learning (ML) algorithms. These opt for either a user-agnostic modeling approach, making the model susceptible to a larger degree of noise, or a personalized approach, where training on individual data alludes to a more limited data set, giving rise to overfitting, therefore, ultimately, having to seek a trade-off by choosing 1 of the 2 modeling approaches to reach predictions.

**Objective:**

The objective of this study was to filter, rank, and output the best predictions for small, multimodal, longitudinal sensor data using a framework that is designed to tackle data sets that are limited in size (particularly targeting health studies that use passive multimodal sensors) and that combines both user agnostic and personalized approaches, along with a combination of ranking strategies to filter predictions.

**Methods:**

In this paper, we introduced a novel ranking framework for longitudinal multimodal sensors (FLMS) to address challenges encountered in health studies involving passive multimodal sensors. Using the FLMS, we (1) built a tensor-based aggregation and ranking strategy for final interpretation, (2) processed various combinations of sensor fusions, and (3) balanced user-agnostic and personalized modeling approaches with appropriate cross-validation strategies. The performance of the FLMS was validated with the help of a real data set of adolescents diagnosed with major depressive disorder for the prediction of change in depression in the adolescent participants.

**Results:**

Predictions output by the proposed FLMS achieved a 7% increase in accuracy and a 13% increase in recall for the real data set. Experiments with existing SOTA ML algorithms showed an 11% increase in accuracy for the depression data set and how overfitting and sparsity were handled.

**Conclusions:**

The FLMS aims to fill the gap that currently exists when modeling passive sensor data with a small number of data points. It achieves this through leveraging both user-agnostic and personalized modeling techniques in tandem with an effective ranking strategy to filter predictions.

## Introduction

### Background

Mobile and wearable sensing has garnered increasing interest in areas of physical health [1,2], mental health [3-5], and activity recognition [6,7]. Multimodal passive sensing accommodates data collection without disrupting the human routine, allowing it to be an important tool to understand human behavior. However, passive sensing, unlike other forms of data, encounters common fundamental challenges in mobile health studies pertaining to physical and mental health. These challenges include small data sets, noisy or sparse data, and sensor selection criteria. Next, we explain these challenges and discuss how our framework can help in alleviating them.

One of the primary challenges in passive sensing studies is small data sets. These arise due to limitations in the sample size of participants, the study duration, and ground truth restrictions. In this study, we explored this challenge from the viewpoint of studies conducted on passive sensing. Studies related to physical health (eg, [1,2]) have investigated dietary behavior with the help of passive sensing. Participant sample sizes in Rabbi et al [1,2] were 17 and 16, respectively, which is a limited participant count. This type of data limitation is even more prominent in mental health research that relies on passive sensing. Studies on depression [3] and schizophrenia [4], for example, had participant sample sizes of 28 and 5, respectively. The limited data sets in passive sensing research are also a factor of the study duration. To understand this, we can observe the duration of study. For example, the study duration in Rabbi et al [1,2] was 21 and 98 days, respectively, while the study by Canzian and Musolesi [3] lasted for 70 days and that by Difrancesco et al [4] was limited to only 5 days. The limitation in data led researchers away from using complex deep learning (DL) models, as demonstrated in previous studies [1-4]. This is because DL models have more hyperparameters and succumb to overfitting due to memorization of the data the models are trained on [8]. In this study, we took inspiration from the existing work and selected specific traditional machine learning (ML) algorithms that are less susceptible to overfitting in small-data scenarios. However, unlike previous studies [1-4,9-17], we also ensured that our predictions were ranked based on 2 different modeling paradigms that further helped circumvent overfitting and also assisted in noise removal, as explained later.

The second challenge commonly faced when tackling passive sensor data is that of sparsity or noise. This challenge arises due to signal inconsistencies and noise in sensor data collection because of software issues, data sync, or hardware problems. Discussions of sparsity and the negative effect it has on modeling have been previously documented [7,18-20]. These studies have presented an overview of the passive sensing landscape and highlighted the role signal inconsistencies can play in predictive modeling of passively sensed data. The fact that data are noisy, especially in the case of wearable sensors, was mentioned by Plötz [18]. Cornet and Holden [19] reported that a lack of sensor precision leads to sparsity, and Xu et al [20] documented the level of noise in data that prevents user-agnostic models from generalizing well. Our proposed framework attempts to reduce the effect of noise by forming a balance between predictions from user-agnostic modeling paradigms and personalized modeling paradigms. In addition, choosing specific ML algorithms, such as Extreme Gradient Boosting (XGBoost), Adaptive Boosting (AdaBoost), elastic-net, and extra-tree, and ranking predictions from them help lessen the impact of sparsity [21-24].

Sensor selection is the third type of challenge that has not received significant attention in passive or mobile sensing literature. Studies have tested various feature combinations mainly in the light of performing feature selection or feature reduction [25]. Joshi and Boyd [26] and Altenbach et al [27], for example, used heuristic-based convex optimization to select sensors from an array of sensors. However, both these studies were purely from the perspective of sensor placement. They did not investigate which combination of sensors provided the best outcome for prediction-based modeling and were more in favor of wireless sensor network establishment. Mobile or wearable devices are laced with multiple sensors, and building and knowing which sensors create optimum models are vital particularly to mental and physical health–related studies. Through our framework, we present a way to test combinations of sensor data and derive and rank predictions from among those combinations, allowing investigators to understand which combinations of sensor data yield the best predictions for their passive sensing experimental setup.

All the aforementioned challenges are common to passive sensing data sets. However, they exhibit significant presence in mental and physical health–related studies [3,4]. Xu et al [20] talked of the general sequence of steps researchers take to build models and the struggles of working with passively sensed data. A strong framework to yield the best predictions can prove to be beneficial to the community at large and bring about greater insight from studies conducted with small data sets.

In this paper, we present our ML modeling and ranking framework to address these challenges. The framework is designed to induce improved predictions for multimodal sensing. It balances both user-agnostic and personalized modeling of small data sets encountered often in mental and physical health–based studies. Our framework makes the following contributions: (1) prediction filtering and ranking through tensor-based aggregation of small, multimodal sensing data sets, (2) sensor combination selection to derive the best predictions, and (3) a reduction in overfitting predictions due to limited data and noise through ensembling of user-agnostic and personalized modeling strategies.

Importantly, it should be noted that by the size of the data set, we refer to the final data sets where raw sensor readings are aggregated into intervals to align with the sampling frequency of ground truth data. In this work, we defined small data sets as those comprising fewer than 1000 data points for training ML models. Sparse or noisy data sets were those that either consisted of many zero entries or data sets for which highly varying sensor values were observed among different participants in the study.

We evaluated the framework through its performance in the context of predicting changes in depression severity in a group of adolescent patients. The results showed the framework’s ability to use multiple modeling approaches for providing robust predictions in critical cases, such as mental health.

Passive sensing data for human behavior modeling are different from other data formats, such as images, audio, or normal tabular data. Researchers in the field of passive sensing agree that passive sensing data have some common properties, such as they are time series data, multimodal, longitudinal, nonlinear, and noisy, as previously discussed [20]. Xu et al [20] also emphasized the researcher’s need for tools that can help ease the time lost in traversing the common pitfalls of passively sensed data. Our work endeavors to resolve such pitfalls for cases where passive sensing data are limited. Next, we discuss the related work highlighting the state of the art (SOTA) in passively sensed small, multimodal data sets.

### Related Work

Despite the growing body of work using multimodal passive sensing in physical and mental health applications [28-32], there exists scope for improvement in small-data scenarios.

In this section, we underline what exists in the current SOTA and why we need a ranking-based framework to address scenarios with small data sets. Keeping in line with our contribution, it will prove beneficial to present the current SOTA through understanding:

- How traditional ML algorithms are applied in the context of passive sensing
- Why complex DL models do not work well in limited data scenarios
- How ensemble modeling has been adapted in passive sensing studies
- What the role of data fusion is in modeling passive sensing data

#### Traditional Machine Learning Algorithms Applied in Passive Sensing

Traditional ML algorithms have been applied to passive sensing in the space of human activity recognition (HAR) [9-11], general health [12-15], and mental health [3,16,17]. A deeper dive into the studies reveals some common takeaways that include the following:

- All of them test multiple ML algorithms, followed by selecting predictions based on the overall chosen validation metric.
- They all follow a singular modeling strategy, resorting to either user-agnostic or personalized modeling.
- Cross-validation (CV) is either K-fold or leave-one-out CV.

This is a repetition of steps that authors in the field make independently and is discussed extensively in the highlighted literature presented in Table 1. Following a single modeling strategy is restricting as choosing to follow a user-agnostic approach exposes the model to a greater degree of noise due to the heterogeneity in sensor values among participants, while solely following a personalized approach reduces data availability further as the model learns from individuals’ data rather than the general population data. Our endeavor through this ranking framework is to combine both the approaches, while using traditional ML algorithms.

**Table 1:** Summary of SOTA^a^ literature using traditional ML^b^ for passive sensing, with special focus on CV^c^, the overall modeling strategy, and ML algorithms.

Study	Application	CV	Modeling strategy	ML algorithm
Kwapisz et al [9]	HAR^d^	10-fold	User agnostic	DT^e^, LR^f^, MLP^g^
Shukla et al [10]	HAR	5-fold	User agnostic	KNN^h^, SVM^i^
Chen and Chen [11]	HAR	10-fold	User agnostic	RF^j^, SVM, KNN
Huang et al [12]	Sleep	10-fold	User agnostic	SVM
Montanini et al [13]	Sleep	K-fold/leave 1 out	User agnostic/personalized	KNN, DT, RF, SVM
Teng et al [14]	Parkinson’s tremors	5-fold	User agnostic	XGBoost^k^, DT, RF
Azam et al [15]	Breath	K-fold	User agnostic	SVM
Canzian and Musolesi [3]	Depression	Leave 1 out	User agnostic	SVM
Grunerbl et al [16]	Bipolar disorder	K-fold	User agnostic/personalized	NB^l^, KNN, DT
Saeb et al [17]	Depression/anxiety	10-fold	User agnostic	XGBoost, DT

^a^SOTA: state of the art.

^b^ML: machine learning.

^c^CV: cross-validation.

^d^HAR: human activity recognition.

^e^DT: decision tree.

^f^LR: linear regression^.^

^g^MLP: multilayer perceptron^.^

^h^KNN: K-nearest neighbor^.^

^i^SVM: support vector machine.

^j^RF: random forest^.^

^k^XGBoost: Extreme Gradient Boosting^.^

^l^NB: naive Bayes^.^

#### Limitation of Deep Learning in Small-Data Scenarios

A common replacement for traditional ML algorithms is DL. Here, we explain why DL models are not ideal solutions for the problem addressed in this study. DL models have gained immense popularity in the literature [33]. Their power lies in modeling the nonlinearity and noisy nature of passively sensed data. DL has a toolkit of strategies to handle small data that includes data augmentation [1], transfer learning [19], and ensembling [29]. However, the size of a small data set in DL studies ranges from 1000 to 10,000 training points [18]. This is unlike the ranking framework presented in this paper, which has been designed for data sets with fewer than 1000 data points. Therefore, despite their superiority in modeling larger passive sensing data sets, the performance of DL models suffers in cases where study data are limited and in the hundreds. The complexity of DL models results in overfitting to small data sets [14]. In this paper, we worked to solve the problem of limiting data by providing researchers with a reproducible way to run multiple models and select the best predictions from among them. By using traditional ML in conjunction with ranked predictions from user-agnostic and personalized models, the issue of overfitting due to model complexity is dealt with in the proposed work.

#### Ensemble Learning to Build Robust Models for Passive Sensing Data

Among the different ways of dealing with overfitting, ensemble learning has been instrumental. Ensemble ML is a widely used approach in passive sensing studies [14,17,34,35]. It mainly exists in the form of boosting [6,14,17,34], bagging [14,16], weighted ensembles [35], and max voting [36] ML algorithms. Ensemble learning presents better results in terms of evaluation metrics. Ensemble learners are trained using a single modeling strategy. Therefore, they are either personalized ensembles [35], which allows learners to derive interesting artifacts at personal levels, or user-agnostic ensembles [14,17,34,36-38], which only generate macrolevel information. Our contribution through the ranking framework is to provide a balance of both macrolevel patterns and user-specific patterns through a weighted ensemble of both approaches. Ensembling in this manner will allow us to reduce the noise that is picked up due to varying sensor values among users and account for user-specific patterns through the predictions on personalized data.

#### Role of Data Fusion in Passive Sensing Studies

**Figure 1:**
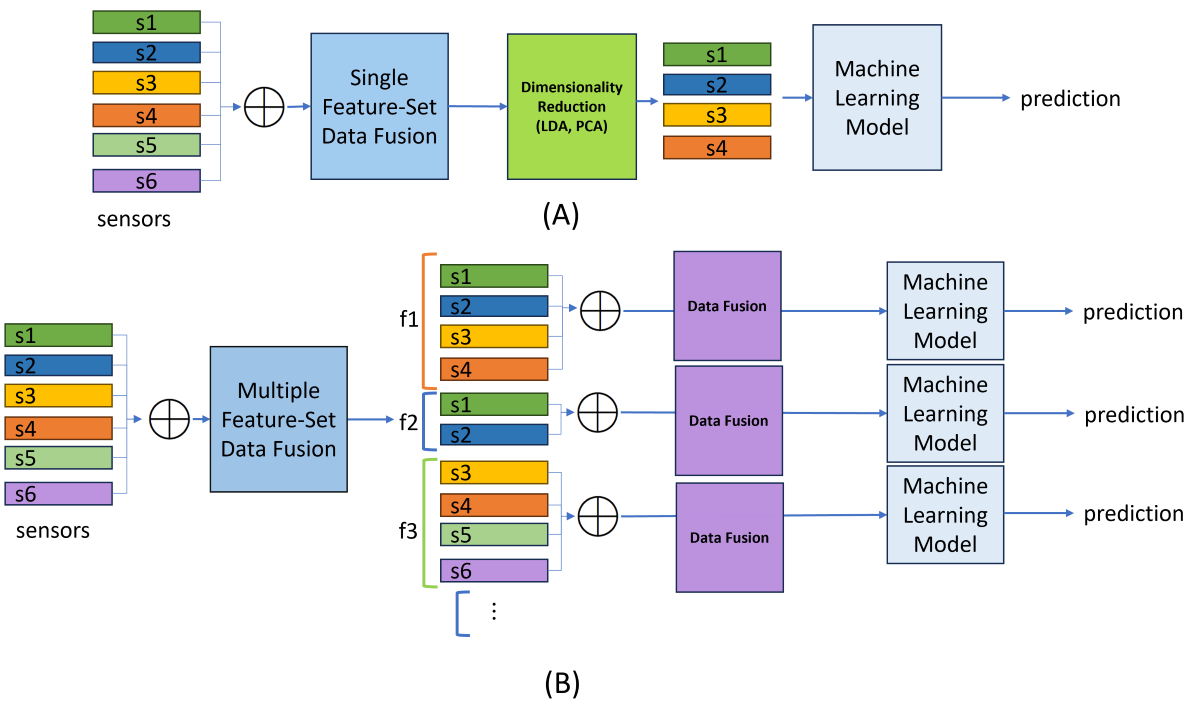
(A) Data fusion approach in the current literature and (B) proposed FLMS data fusion approach, where s1-s6 represent distinct sensors and f1-f3 represent feature set combinations, which were then fused prior to ML modeling. FLMS: framework for longitudinal multimodal sensors; LDA: linear discriminant analysis; ML: machine learning; PCA: principal component analysis.

The use of data fusion in passive sensing has seen a steady growth due to the use of multimodal sensors in passive sensing studies. Earlier studies were often restricted to single sensors, which were then manipulated to obtain a handful of features. For example, Canzian and Musolesi [3] primarily used GPS sensor data, while Kwapisz et al [9] only opted for an accelerometer to base their predictive modeling. The way data fusion is approached has a common link among the surveyed studies in the current literature. The studies have applied feature-level fusion [10,39-43], where fusion takes place after feature extraction from raw signals. A single feature set is generated and then passed on to dimensionality reduction, such as linear discriminant analysis (LDA) [10] or principal component analysis (PCA) [40-42]. The focus in these papers tends to be a reduction in dimension, without trying to study the impact of multiple distinct feature combinations. In comparison, our contribution of feature selection focuses on studying the relationship between each group of sensors by creating multiple feature sets based on sensor availability. This will allow us to select the best set of features to work with for a specific type of study. An illustration of the difference in the existing literature and our feature fusion approach is shown in Figure 1 [10,39-43].

Overall, our ranking framework is motivated to aid researchers in situations in which data sets are small, sparse, or noisy and multimodal by taking advantage of its multiple model generation and the balanced outcome of the best predictions.

## Methods

### Ethical Considerations

The data collection was approved by the Institutional Review Board of the University of Pittsburgh Human Research Protections Office (STUDY18120176).

### Data Description

The study used passive sensing data and is presented through the lens of depression change prediction among adolescents. The data set comprised 55 adolescents from 12 to 17 years old, with an average age of 15.5 (SD 1.5) years. The AWARE app was used to collect the participants’ smartphone and Fitbit data. The data completeness rate for AWARE and Fitbit was, on average, 65.11% and 30.36%, respectively. The levels of completeness echoed the difficulty in collecting passive sensing data. Smartphone and Fitbit data were collected from each participant over 24 weeks.

The 9-item Patient Health Questionnaire (PHQ-9) [44] was used to collect weekly self-reports of depression severity from the participants. The questionnaire consists of a set of 9 questions, which can be scored from 0 to 3, giving a score range of 0-27. We used PHQ-9 scores as the ground truth to compare the prediction accuracy of our models.

#### Relation of Sensor Data to Mental Health

Raw sensor data, including calls, location, conversation, screen usage, Wi-Fi, steps, sleep, and heart rate, were processed, and relevant features were extracted at daily intervals. We used RAPIDS [45] to extract 72 features from the sensors. The existing literature [3,46-51] shows how location [3,46,49,50,52], calls [48,53], screen usage [46,54,55], conversations [55-58], Wi-Fi [48,59], steps [60], and heart rate [61] can be effective in predicting mental health behavior. Studies [3,46,49,50] have used location sensors, such as the GPS, and shown a strong relation to depressive symptom severity. Clinical measures, such as the PHQ-9 [44], the PHQ-8 [62], the Hamilton Rating Scale for Depression (HAM-D) [63], and the Hamilton Rating Scale for Anxiety (HAM-A) [64], have been used as target labels for prediction using sensor-based features, establishing a proof of association between sensor features and mental health predictions. Studies [47,48,51,54,60] have used multimodal sensors of smartphones that included the sensors we chose for this study: calls, location, conversation, screen usage, Wi-Fi, Fitbit steps, and Fitbit heart rate. In the *Results* section, we further elaborate on the feature engineering from each of the sensors. The validity of using the sensors to predict mental health, in particular the choice of sensors, was motivated by the aforementioned studies, which showed strong predictive capability of sensors in the area of mental health prediction.

### Framework Design and Modeling

We proposed a framework for longitudinal multimodal sensors (FLMS) as a ranking framework to rigorously handle longitudinal, multimodal sensor data and incorporate different analysis and modeling strategies suited for small and sparse time series data sets to produce better results. The FLMS incorporates 4 stages to improve, rank, and filter data set predictions (see Figure 1):

- Stage 1: multimodal sensor fusion to explore the data set from multiple views and to identify the minimum number of sensors necessary to yield a good prediction. It also addresses sparsity.
- Stage 2: ML modeling with combined user-agnostic and personalized approach. This stage is designed to leverage user-agnostic and personalized predictions. The ML algorithms used in this stage were chosen due to their superior prediction capability in small-data scenarios and their ability to tackle sparse data sets.
- Stage 3: tensor-based aggregation and ranking leverage predictions from all fused combinations and modeling strategies to calculate more robust predictions.
- Stage 4: final prediction informed by the ensemble weighted average of both user-agnostic and personalized predictions to reduce the effect of overfitting in small data sets. This stage uses weights calculated via hamming distances to prevent any modeling approach from dominating the predictions.

**Figure 2:**
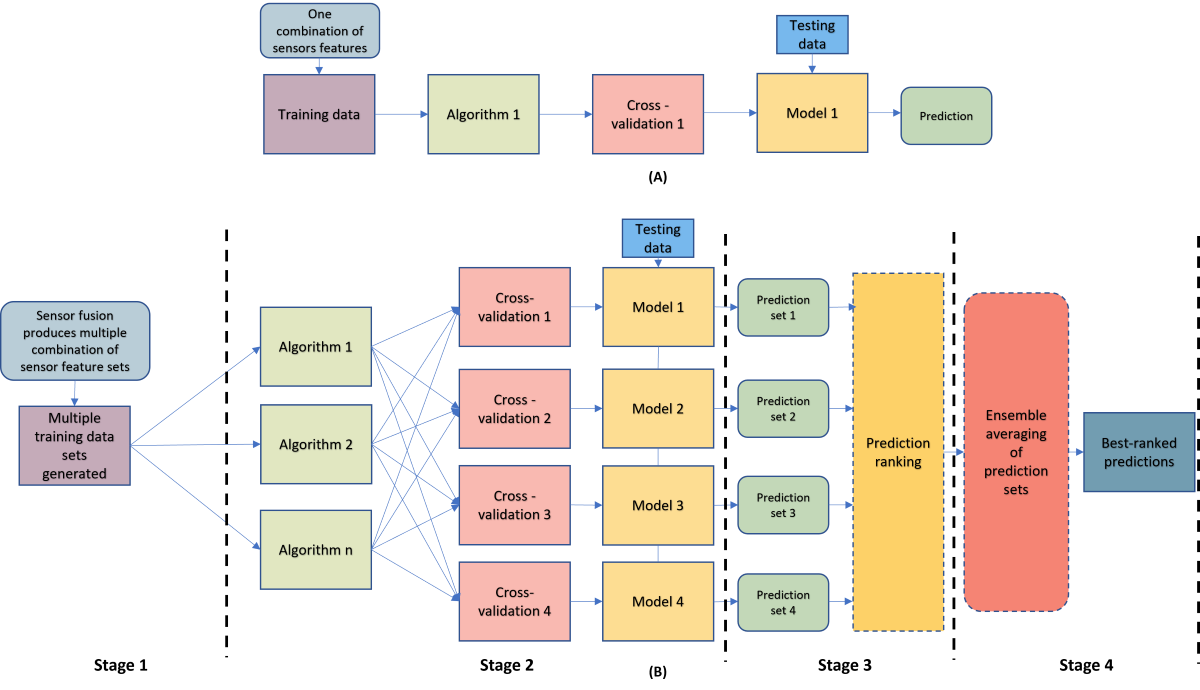
(A) Conventional modeling approach and (B) proposed FLMS approach. FLMS: framework for longitudinal multimodal sensors.

A high-level view in Figure 2 illustrates how the FLMS is different from conventional ML approaches. Observing Figure 2A, we understand that the conventional modeling strategy uses a single algorithm with either a user-agnostic CV, where all users are included in the training and test sets, or a personalized CV strategy, where a single user’s data are used to derive predictions. However, Figure 2B displays how the FLMS uses different combinations of sensors as input data, followed by multiple algorithms and a combination of user-agnostic and personalized modeling. The modeling stage is followed by a ranking of predictions and finally an ensemble of the predictions to yield the final output.

A detailed explanation of the stages of the FLMS and their utility is provided next.

#### Stage 1: Multimodal Sensor Fusion

Stage 1 was designed for the early fusion of sensors at a feature level. Sensor fusions followed a combinatorial approach using 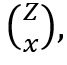, where Z is the total number of modalities available and x is the number of sensors to fuse. Our case study had 6-sensor modalities that generated a set of 63 separate data sets calculated as 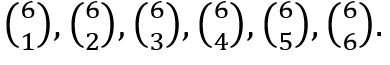.

Data set preprocessing steps involved normalization and log transforms. Imputations to fill missing feature observations were also conducted. The framework allowed for implementation of the K-nearest neighbor (KNN) algorithm for imputation, which is also the first level of defense against sparsity. The generated data sets were in 2D tabular data format. The sensor data were aggregated according to the granularity of the ground truth. Our case study collected PHQ-9 scores as an accepted depression measure. The total score range of the 9 questions was 0-27. This was collected on a weekly basis, and thus, our daily data were aggregated in weekly intervals.

#### Stage 2: ML Modeling With a Combined User-Agnostic and Personalized Approach

Stage 2 focused on modeling and predictions based on the data sets generated in stage 1. All stage 1 data sets were run through the modeling suite, which encompasses a series of ML algorithms and CV strategies to help build user-agnostic and personalized models.

The ML suite includes case-specific linear and nonlinear algorithms. For our case study on adolescent depression, we followed a regression-based approach, and therefore, we selected algorithms such as linear regression (LR), elastic-net, random forest (RF), AdaBoost, extra-tree, gradient boosting, and XGBoost. The algorithms were chosen based on (1) their performance in the existing literature when working with small data and robustness to sparsity, and (2) tree-based models, which were specifically chosen to provide added tractability for researchers to inspect which features mainly contributed to the models’ predictive capability. The algorithms were used in each modeling strategy. The predictions of the ML algorithms for each time unit were stored in arrays for each participant and later used to select the best model for each participant. The best model selection strategy chose the model with the minimum error (in the case of regression) or the maximum accuracy (in the case of classification) among all algorithms. For example, among l number of regression algorithms, the best model was chosen as follows:



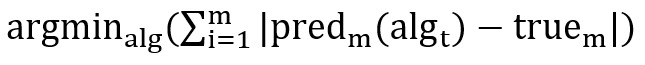



**(1)**

,where alg refers to the algorithm with the lowest absolute sum error and pred_m_(alg_t_) is the prediction made by an algorithm l at unit time t. The array of prediction by the best model was retained for each respective participant.

#### User-Agnostic Model Building

**Figure 3:**
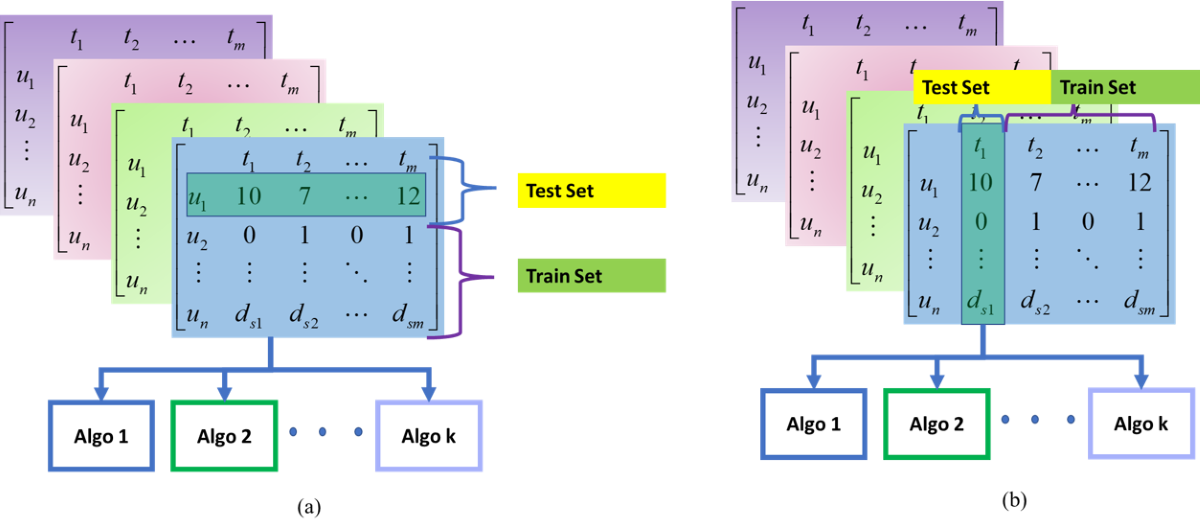
User-agnostic model building: (A) LOPO and (B) LTXO strategies. Algo: algorithm; LOPO: leave one participant out; LTXO: leave time unit X out.

To leverage as much data as possible, we implemented the leave-one-participant-out (LOPO) and leave-time-unit-X-out (LTXO) strategies. This is illustrated in Figure 3A,B.

In LOPO, we held out all data from a single participant for validation and trained the model on other participants. This strategy reflected the cold start case where a new user started using the health app.

The LTXO is based on the unit of time for ground truth data (eg, a week). For training, we held out a given time unit of all participants and trained the model on the rest of the time units. This strategy evaluated the impact of time-specific segments of data on prediction. The training phase captures the similarity and variation of data during different time units to build user-agnostic models.

#### Personalized Model Building

The personalized modeling strategy leverages each user’s historical and cross-time data samples in a sliding window and the leave-one-time-unit-out approach.

For each participant, the accumulated-time-unit (ATU) strategy built a model from X_t_ time units of data to predict X_t+1_. For example, the model built from weeks 1 and 2 predicted depression in week 3. In the next iteration, the sliding window was increased by T time units (eg, 2 weeks) to repeat the model-building process. This process continued until the maximum number of time units was reached. This method examined the forecasting capability of the framework.

**Figure 4:**
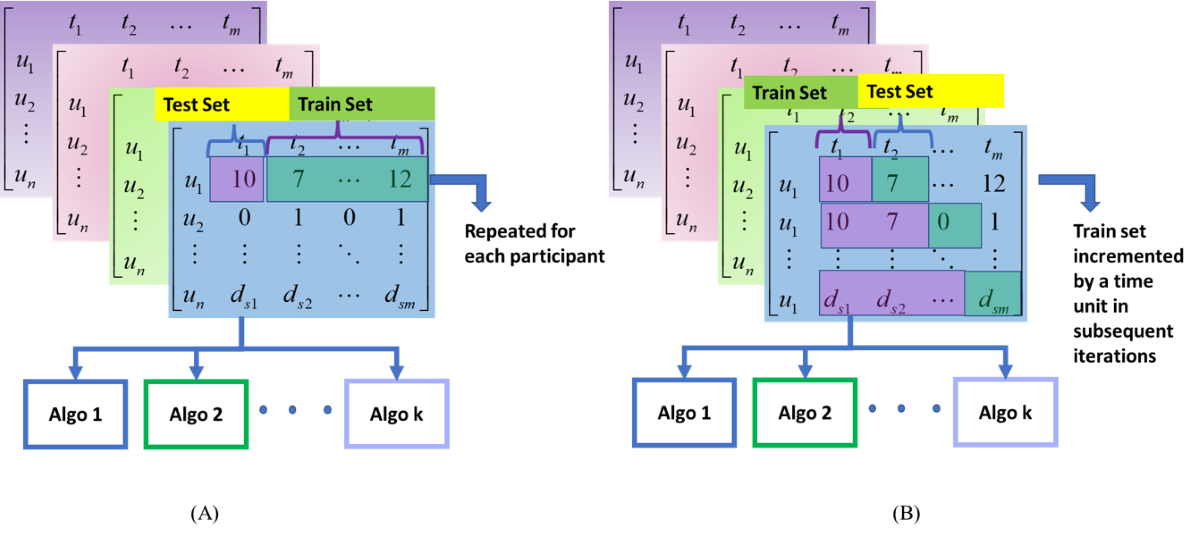
Personalized model building: (A) LOTPO and (B) ATU strategies. Algo: algorithm; ATU: accumulated time unit; LOTPO: leave one time unit of participant out.

The leave-one-time-unit-one-participant-out (LOTPO) strategy trained the models on all time units of a participant across time to predict the target label for the current time unit. For example, for a participant with 10 weeks of data, we built a model from data in weeks 1-5 and weeks 7-10 to predict depression in week 6. This method evaluated the feasibility of past and future data for each participant to predict an outcome (Figure 4A,B).

#### Stage 3: Tensor-Based Aggregation and Ranking

**Figure 5:**
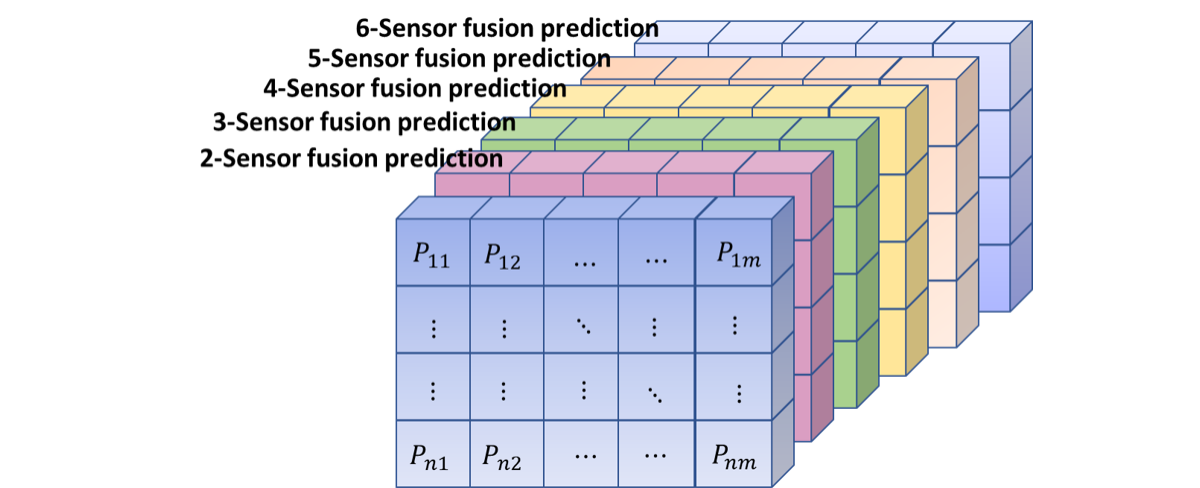
An example of tensor representation of 6-sensor fusion predictions.

The output of stage 2 was a set of best prediction matrices for sensor fusion combinations, where each slot in the matrix represented prediction results for a participant in a particular time unit. We represented these predictions in the form of Z-dimensional tensors (Figure 5), where Z is the number of modalities being used. For example, a study with 6 modalities and 45 users over 24 weeks was represented in tensor form as (6, 45, 24). The tensor representation helped represent the high dimensionality of sensor combinations.

The predicted values for each slot across tensors were then aggregated using an aggregation function (eg, mean). This process took advantage of the stage 2 combinations to help reduce the error in prediction. For example, we aggregated predictions of 6 tensors (generated from 5-sensor fusion) into 1 tensor by calculating the mean of the predictions from the 6 combinations (see Figure 3). This was done for both user-agnostic and personalized models. The aggregated mean was calculated using the following equation:



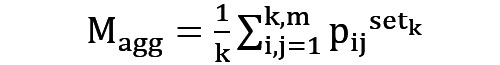



**Figure 6:**
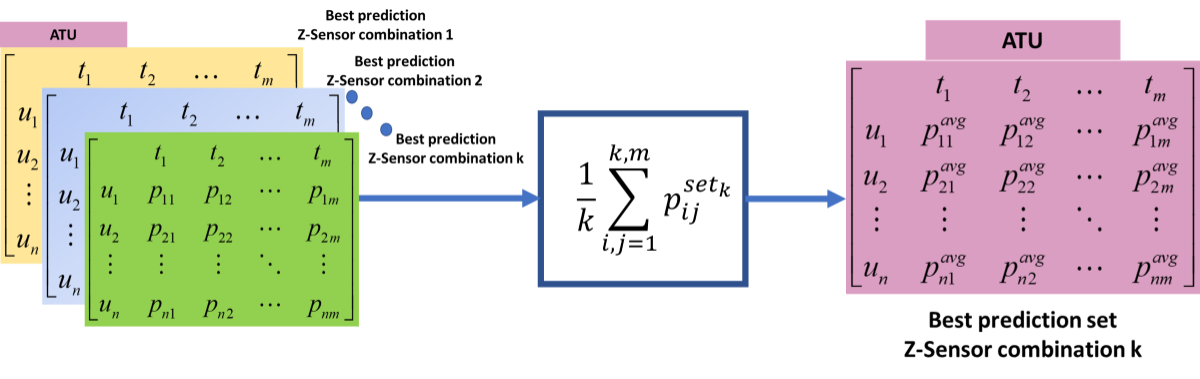
Instance of ATU where it shows how the mean aggregated prediction set is generated according to Equation (2). ATU: accumulated time unit; avg: average.

**(2)**

,where M_agg_ is the aggregated mean, k is the total number of sensor combinations aggregated, i is the combination number, j is the corresponding time unit, and 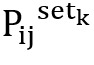 is the prediction across each set of combinations. The data were now in a format where each 2D tensor represented a particular sensor fusion prediction set (Figure 6).

**Figure 7:**
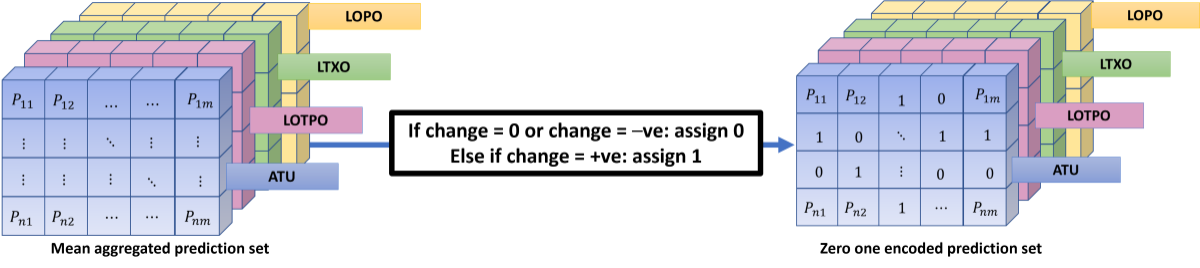
The 0-1 encoding process resolves dealing with large variances in regression values. ATU: accumulated time unit; LOPO: leave one participant out; LOTPO: leave one time unit of participant out; LTXO: leave time unit X out.

The predictions were next encoded into 0s and 1s to counter the large variance in the regression values from the original values. This logic can be set based on the type of ML problem the framework is being used to address. For example, in our case study, if the regressed change in depression score values was 0 or negative value, we classified it as 0, and if it was positive, we represented it as 1 (Figure 7).

**Figure 8:**
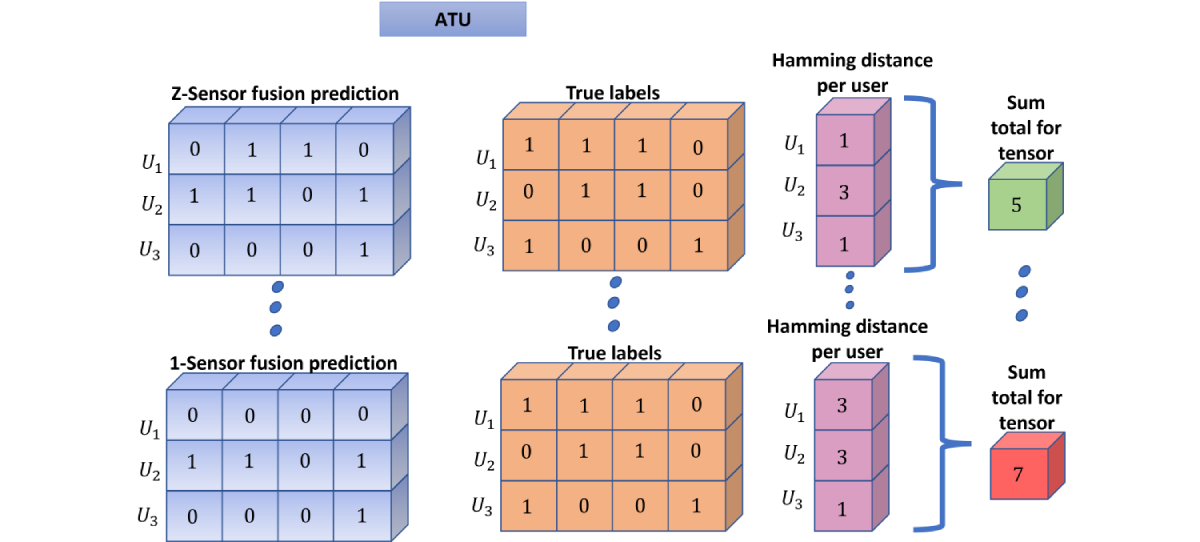
Hamming distance calculations reduce error and also determine the weight each of the 4 modeling approaches will contribute to stage 4’s ensembled weighted average. ATU: accumulated time unit.

The next step in this stage measured the hamming distance between the 0-1‑encoded tensor and the true labels tensor, as shown in Figure 8. These hamming distances were then aggregated (D_u_) for the respective 2D tensor as follows:



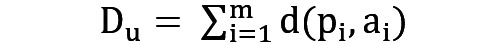



**(3)**

,where d(p_i_, a_i_) is the hamming distance between unit time predictions p_i_ and the true value a_i_. Based on the measured distance, we ranked and chose the best set of predictions. This metric helped inform the choice of weightage to associate with a particular modeling strategy. The hamming distance helped further reduce errors after encoding and filtered down to the best set of predictions from each strategy.

#### Stage 4: Weighted Ensemble

**Figure 9:**
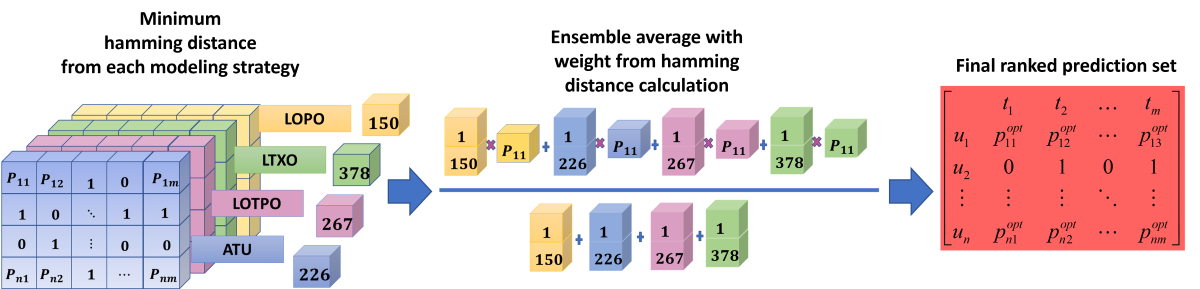
Ensemble average based on weights derived from the hamming distance to arrive at best-ranked predictions. ATU: accumulated time unit; LOPO: leave one participant out; LOTPO: leave one time unit of participant out; LTXO: leave time unit X out.

The final stage formed the most robust set of predictions via an ensemble weighted average approach, where weights were calculated based on the minimum hamming distances derived from each modeling strategy in stage 3 (Figure 9):



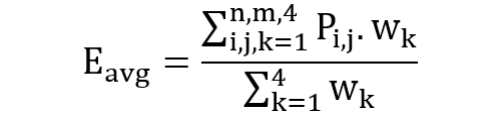



**Figure 10:**
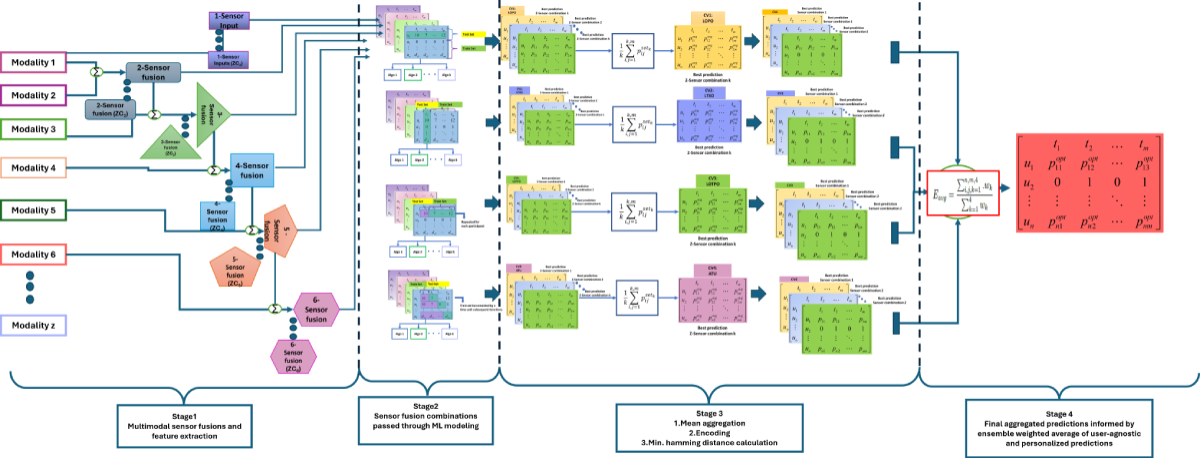
FLMS ranking overview. Algo: algorithm; ATU: accumulated time unit; avg: average; CV: cross-validation; FLMS: framework for longitudinal multimodal sensors; LOPO: leave one participant out; LOTPO: leave one time unit of participant out; LTXO: leave time unit X out; ML: machine learning.

**(4)**

,where P_ij_ is the prediction tensor, w_k_ is the weight based on the minimum hamming distance, and i and j are the number of users and time units, respectively. The data were then encoded back to 0s and 1s. A complete version of the FLMS with all its stages is presented in Figure 10 (see Multimedia Appendix 1 for a higher quality image). 

## Results

### Stagewise Description of Framework Processing on an Adolescent Data Set

To evaluate the performance of the proposed FLMS, we used a depression data set of adolescents. This was a small data set, comprising noisy, multimodal sensor values from multiple participants—a suitable case study for our purpose of evaluating the performance of our proposed framework. Before presenting the experimental results, we first provide an understanding of how the adolescent data set was processed at each stage of the FLMS.

The passively sensed depression data set was longitudinal, with a varying number of observations per participant. The goal was to predict changes in the depression score. This was achieved by passing the small set of observations through our ranking framework, which processed, modeled, ranked, and output the best set of overall predictions based on multiple modeling approaches. A prediction of change in depression is difficult and becomes even more challenging when the amount of data provided to the ML algorithms is limited.

#### Stage 1 Outcome

As part of stage 1, daily data were aggregated in weekly intervals to align with weekly ground truth values. Based on our extensive exploratory data analysis (EDA), we set thresholds for sparsity and adopted KNN as the imputation strategy.

Our final data set consisted of 507 data points with 72 features, with an average of 13 weekly data points per participant. A series of data sets were then produced from an early fusion of 6-sensor features. Each data set retained 45 (81.8%) of the 55 participants. We had to drop 11 (20%) participants as they were missing more than 60% of their sensor data. The true depression state of the participants was given by the PHQ-9 weekly survey. The change in participant depression scores was calculated as W_m_ – W_m–1_, where W_m_ is the score for the m-th week; this served as the ground truth for our analysis.

#### Stage 2: ML Modeling Outcome

The ML algorithms in stage 2 regressed on the change in the depression score, with positive changes exhibiting a rise in the depression score in that week, negative changes representing a decrease, and 0 marking no change. The best predictive models of depression for each participant were built and selected following the steps in stage 2.

#### Stage 3: Encoding and Prediction Filtering Outcome

This led to stage 3, where after the mean aggregation, we encoded the regressed values as our goal was to predict whether the change in the depression score was positive, negative, or constant, rather than determining the exact value of the change. This step was followed by hamming distance calculations to further rank and filter the best set of predictions.

#### Stage 4: Final Prediction Ensembling of Adolescent Data

The predictions evaluated by the minimum hamming distances entered stage 4, where we calculated the final ensemble predictions. The predictions used weights determined by hamming distance calculations, which enabled us to balance between personalized and user-agnostic models. This step completed the offline training and prediction of change in depression in the adolescent data set.

### Experiment Design and Results

In this section, we present the depression change prediction results of the FLMS. The experiments were designed to test the framework’s claims of reducing overfitting on a small data set, reducing the impact of noise or sparsity, and identifying the best combination for sensor fusion.

We conducted 3 main experiments in support of our claims:

- Experiment 1 tested FLMS predictions against singular modeling strategies used in SOTA. This experiment evaluated our claim regarding the advantage of the overall framework that took steps to reduce noise and identify the best sensor combinations versus a singular modeling strategy.
- Experiment 2 was a SOTA comparison test conducted to evaluate how our prediction-ranking framework performed in comparison to existing ML and DL approaches used in the current literature. This comparison also substantiated the FLMS performance to overfitting versus the existing strategies in the literature from prediction in small-data scenarios.
- Experiment 3 was designed to compare the FLMS performance with that of commonly used ML algorithms that have been shown to perform well with sparse data. It is important to note that there is an overlap of ML algorithms used to tackle sparsity and those used in passive sensing studies for mental health, particularly for small data sets.

#### Evaluation Metrics

The task of the FLMS is to model, rank, and output the best set of predictions from multiple modeling approaches. The output of the FLMS are predictions encoded as 0s or 1s (ie, binary values). Therefore, our choice of evaluation metrics for the framework predictions was the average accuracy, average recall, and average *F*_1_-scores amongst users.

#### Experiment Metadata

The metadata pertaining to each experiment is provided at the end of the experiments. The information included as metadata is based on the best practices used [65] to help with reproducibility of results. They include (1) feature preprocessing steps, (2) modeling CV strategy, (3) ML algorithms used, (4) random state, and (5) evaluation metrics specific to the experiments. They are presented in the form of tables following the corresponding results for each experiment.

#### Data Set Used in the Experiments

To standardize our experiments, we maintained a consistent data set, a combination of 6-sensor feature sets that included calls, location, screen usage, conversation, Fitbit, and Wi-Fi. After the stages of preprocessing, missing data imputation using the KNN strategy, and the removal of highly corelated features, the final data set comprised 61 features and 507 data points belonging to a total of 45 (81.8%) participants.

#### Feature Engineering in Experiments

Since we maintained a consistent data set for all our experiments, feature engineering for all the experiments was achieved through data collected from 6 sensors. As discussed earlier, the data were collected from participants’ smartphones using the AWARE app [66] and then passed through the RAPIDS application programming interface (API). The features extracted using the API are discussed in detail next.

##### Call Sensor Features

The calls sensor features provide a context of how frequently the user has been in contact with someone else. Studies have revealed that higher degrees of depression are linked to reduced contact with social circles [48,53]. As part of call sensor features, we extracted the total number of missed calls; the counts of missed calls from distinct contacts, calls from the most frequent contacts for a time segment, incoming calls, and outgoing calls; the mean (SD), maximum, and minimum duration of both incoming and outgoing calls; and the entropy duration of outgoing and incoming calls, which provided an estimate of the Shannon entropy for the duration of all calls of a particular call type (ie, incoming, outgoing, or missed). All the extracted features were mean-aggregated over the period of 1 week to match the ground truth.

##### Location Sensor Features

Location sensor features provide a contextual idea of the amount of movement users of the sensors go through and show the correlation to mental health [3,46,49,50]. The location data are collected through the phones’ GPS or the cellular towers around the phones. Location has been proven to be able to predict depressive states [3]. The features extracted from the location sensors included the location variance calculated through the sum of variance in longitude and latitude coordinates, the log of the location variance, the total distance covered, and the circadian movement [17] calculated using the Lomb-Scargle method that maps a person’s location patterns following the 24-hour circadian cycle. The speed was also captured as a feature, and static labeled samples were clustered and K-means clustering was used to locate significant places visited by the participants. In addition, location entropy was also engineered to provide the proportion of time spent at each significant location visited during a day.

##### Screen Sensor Features

Screen sensor features are a strong indicator of how engaged users are with their phones. To capture this information, we extracted features that includes the minimum, maximum, sum, and mean (SD) of unlock episodes, along with the number of all unlock episodes and minutes until the first unlock episode. These features have been used in prior studies that proved their correlation to depressive symptom severity [46,54,55].

##### Conversation Sensor Features

Conversation is yet another interesting set of features that provide information pertaining to social interactions and has been used in a number of studies relating to mental health [55-58]. The computed features included the minimum, maximum, sum, and mean (SD) of the duration of all conversations. We also recorded the minutes of voice, silence, and noise. The energy associated with noise, which is the L2-norm and the sum of all energy values when noise or voice, was inferred.

##### Fitbit

Fitbit offers 2 features, which we extracted based on their application in previous studies relating to mental health [54,60,61], and included the maximum resting heart rate (average maximum heart rate over 1 week) and the maximum number of steps (average step count over 1 week). These features provided an idea of the physical movement and stress experienced by participants.

##### Wi-Fi

Wi-Fi can be a good indicator of social context. We extracted the Wi-Fi count scans that told us the number of scanned Wi-Fi access points connected to by the phone during a time segment and the number of unique connected devices during a time segment based on the hardware address. In addition, we extracted the most scanned connected device. The use of Wi-Fi-based features in mental health prediction have been previously covered [48,59].

The data set used in our experiments had all the features discussed, which were part of the 61 features. Feature engineering helped provide a context to the data gathered from all the smartphones and Fitbit sensors and form predictions for ML models.

### Results of Experiment 1

Experiment 1 showcased the overall performance of the FLMS in comparison with traditional user-agnostic and personalized models. The FLMS achieved a mean accuracy of 0.66 (SD 0.53) and a mean recall of 0.59 (SD 0.50), which are 7% and 13% higher than the best baseline performance achieved by ATU modeling. Among the singular modeling approaches, the ATU, a personalized strategy, performed best overall, with a mean accuracy of 0.59 (SD 0.50) and a mean recall of 0.46 (SD 0.66). The worst performances were shown by user-agnostic LOPO and LTXO approaches, both of which had a mean accuracy of 0.45 (SD 0.80) and 0.47 (SD 0.83), respectively. These results are presented in Table 2 and show that singular modeling approaches used in different studies [1-4,9-17] underperform when modeling involves small, noisy, multimodal sensor data in comparison to our FLMS. The FLMS uses a balance of these strategies to improve predictions.

Experiment 1 was also designed to show how the FLMS suggests the best feature combinations for the various modeling strategies it uses through the utility of hamming distance from stage 3. The lowest hamming distance in stage 3 for the various modeling approaches used is presented in Table 3. We observed that the ATU approach led to the lowest hamming distance of 226, followed by LOTPO, with a minimum hamming distance of 267. The highest hamming distances were those of LOPO at 350 and LTXO at 378. The lower the hamming distance, the closer the predictions to ground truth. Based on this, we saw that overall, 6-sensor fusion works best for this data set. The metadata of experiment 1 are shown in Table 4.

**Table 2:** Experiment 1 performance of the FLMS^a^ in comparison to singular modeling strategies.

Modeling strategy	Type of modeling strategy	Test accuracy, mean (SD)	Test recall, mean (SD)	Test *F*_1_-score, mean (SD)
FLMS	User agnostic + personalized	0.66 (0.53)	0.59 (0.50)	0.56 (0.55)
ATU^b^	Personalized	0.59 (0.60)	0.46 (0.66)	0.50 (0.57)
LOTPO^c^	Personalized	0.53 (0.65)	0.45 (0.70)	0.32 (0.73)
LOPO^d^	User agnostic	0.45 (0.80)	0.43 (0.72)	0.40 (0.87)
LTXO^e^	User agnostic	0.47 (0.83)	0.35 (0.81)	0.33 (0.86)

^a^FLMS: framework for longitudinal multimodal sensors.

^b^ATU: accumulated time unit.

^c^LOTPO: leave one time unit one participant out.

^d^LOPO: leave one participant out.

^e^LTXO: leave time unit X out.

**Table 3:** Experiment 1 minimum hamming distance for choosing the best sensor combination for the experiment.

Best sensor fusion	Modeling approach in the FLMS^a^	Hamming distance
6-sensor fusion (calls + location + screen usage + conversation + Fitbit + Wi-Fi)	ATU^b^	226
6-sensor fusion (calls + location + screen usage + conversation + Fitbit + Wi-Fi)	LOTPO^c^	267
1-sensor fusion (location)	LOPO^d^	350
2-sensor fusion (calls + location)	LTXO^e^	378

^a^FLMS: framework for longitudinal multimodal sensors.

^b^ATU: accumulated time unit.

^c^LOTPO: leave one time unit one participant out.

^d^LOPO: leave one participant out.

^e^LTXO: leave time unit X out.

**Table 4:** Experiment 1 metadata.

Metadata	Experiment 1
Feature preprocessing	KNN^a^ imputation, dropping highly co-related columns, sklearn StandardScaler
Modeling CV^b^ strategy	FLMS^c^, ATU^d^, LOTPO^e^, LTXO^f^, LOPO^g^
ML^h^ algorithms used	import XGBoost^i^ as xgb sklearn.linear_model import LinearRegression sklearn.ensemble import RandomForestRegressor sklearn.linear_model import ElasticNet sklearn.ensemble import GradientBoostingRegressor sklearn.ensemble import ExtraTreesRegressor sklearn.ensemble import AdaBoostRegressor
Random state	42
Evaluation metrics	Accuracy, recall, *F*_1_-score

^a^KNN: K-nearest neighbor.

^b^CV: cross-validation.

^c^FLMS: framework for longitudinal multimodal sensors.

^d^ATU: accumulated time unit.

^e^LOTPO: leave one time unit one participant out.

^f^LTXO: leave time unit X out.

^g^LOPO: leave one participant out.

^h^ML: machine learning.

^i^XGBoost: Extreme Gradient Boosting.

### Results of Experiment 2

**Figure 11:**
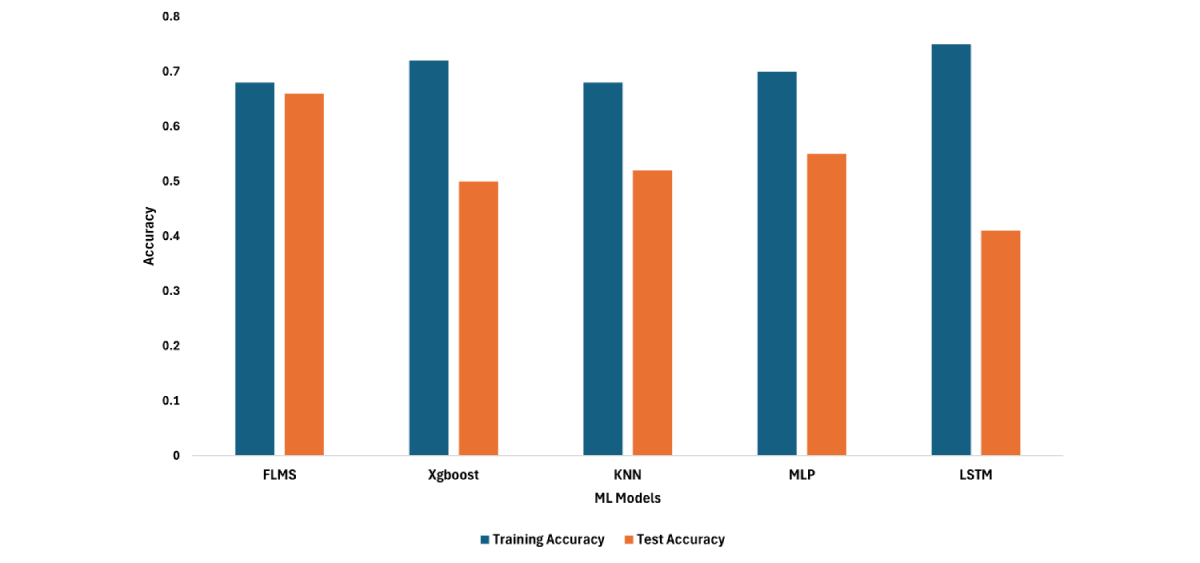
Experiment 2 shows FLMS training and test accuracies in comparison to SOTA models. The FLMS is better at adapting to overfitting compared to the other algorithms. FLMS: framework for longitudinal multimodal sensors; KNN: K-nearest neighbor; LSTM: long short-term memory; ML: machine learning; MLP: multilayer perceptron; SOTA: state of the art; XGBoost: Extreme Gradient Boosting.

In experiment 2, we compared FLMS ranking results with ML algorithms that have been used in multiple studies on sensor-based assessment of mental health, as listed in Table 1. The ML algorithms XGBoost and KNN were chosen based on the popularity of their usage in the community, while the DL algorithm was chosen to be a basic multilayer perceptron (MLP) network and a long short-term memory (LSTM) network. These were also the best-performing algorithms compared to other ML algorithms in the literature on our data set. We initially tried using K-fold validation for the SOTA algorithms, but due to poor results, we switched to the leave-one-out strategy, which performed relatively better. This experiment first compared the overall performance of the FLMS with other SOTA algorithms based on the average test accuracy, recall, and *F*_1_-score. Second, the experiment substantiated the claim that the FLMS is better in tackling overfitting, as shown by the mean training accuracy versus the mean test accuracy compared to the ML algorithms in Figure 11. The models with only the single ML algorithm performed no better than the majority baseline approach, with XGBoost showing a mean test accuracy 0.50 (SD 0.55) and the KNN showing around the same mean accuracy of 0.52 (SD 0.54), as shown in Table 5. The MLP achieved higher accuracy but a low test *F*_1_-score, indicating the model’s performance has high false-positive and false-negative rates. The LSTM was no different and showed a similar recall and *F*_1_-score outcomes. The overfitting of the SOTA models is illustrated in Figure 11, where we compared the FLMS and the rest of the algorithms based on their respective performances using training and test accuracies. Figure 11 shows that the FLMS had a relatively consistent performance between a training accuracy of 68% and a test accuracy of 66%, while XGBoost, KNN, MLP, and LSTM models had high training accuracies but low test accuracies. The metadata of experiment 2 are shown in Table 6.

The experiments demonstrated support for the points highlighted in the contribution of this paper—that our ranking framework works well with small data sets in comparison to existing approaches and can reduce overfitting by using a balance-weighted ensembling of user-agnostic and personalized models.

**Table 5:** Experiment 2 performance of the FLMS^a^ compared to ML^b^ and DL^c^ algorithms used in the current literature on adolescent data.

Predictive learning approach	Modeling strategy	Test accuracy, mean (SD)	Test recall, mean (SD)	Test *F*_1_-score, mean (SD)
FLMS	ATU^d^ + LOTPO^e^ + LOPO^f^ + LTXO^g^	0.66 (0.53)	0.59 (0.50)	0.56 (0.55)
XGBoost^h^ [14,17]	Leave 1 out	0.50 (0.55)	0.33 (0.52)	0.28 (0.57)
KNN^i^ [10,11,13,16]	Leave 1 out	0.52 (0.54)	0.40 (0.61)	0.30 (0.73)
MLP^j^ [9]	Leave 1 out	0.55 (0.70)	0.50 (0.71)	0.33 (0.70)
LSTM^k^ [67]	Leave 1 out	0.41 (0.66)	0.25 (0.70)	0.35 (0.70)

^a^FLMS: framework for longitudinal multimodal sensors.

^b^ML: machine learning.

^c^DL: deep learning.

^d^ATU: accumulated time unit.

^e^LOTPO: leave one time unit one participant out.

^f^LOPO: leave one participant out.

^g^LTXO: leave time unit X out.

^h^XGBoost: Extreme Gradient Boosting.

^i^KNN: K-nearest neighbor.

^j^MLP: multilayer perceptron.

^k^LSTM: long short-term memory.

**Table 6:** Experiment 2 metadata.

Metadata	Experiment 2
Feature preprocessing	KNN^a^ imputation, dropping highly co-related columns, sklearn StandardScaler
Modeling CV^b^ strategy	FLMS^c^, leave 1 out
ML^d^ algorithms used	import XGBoost^e^ as xgb sklearn.neural_network import MLPClassifier sklearn.neighbors import KNeighborsClassifier keras.layers import LSTM^f^
Random state	42
Evaluation metrics	Accuracy, recall, *F*_1_-Score

^a^KNN: K-nearest neighbor.

^b^CV: cross-validation.

^c^FLMS: framework for longitudinal multimodal sensors.

^d^ML: machine learning.

^e^XGBoost: Extreme Gradient Boosting.

^f^LSTM: long short-term memory.

### Results of Experiment 3

Sparsity is a challenge in dealing with small data sets. The large number of 0s or missing values can misdirect models and lead to overfitting [68]. Therefore, it is important to handle the problem of sparsity. Our experiment was designed specifically for small data sets, where sparsity proves to be a challenge. To tackle sparsity in small-data scenarios, the commonly used ML algorithms are KNN, MLP, support vector machine (SVM), decision tree (DT), random forest (RF), XGBoost, and AdaBoost [21-24,69-71].

In our experiment, we showcased a comparison of the FLMS with all the mentioned ML algorithms. We first calculated the sparsity of the adolescent data set that comprised all 6-sensor feature sets. The reason for continuing to use the 6-sensor feature sets as in the prior experiment was to test the algorithms with a data set that had a higher degree of sparsity compared to other feature combinations with lower number of sensors. The sparsity for this data set was calculated as the ratio of 0s to the total number of elements in the data set and is given as follows:



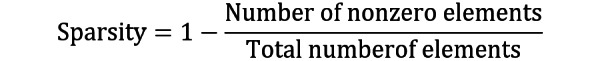



**(5)**

The sparsity of the data set used for this experiment was 35%. In a small data set, this is a significant amount of sparsity to negatively impact ML algorithms.

We performed the modeling and evaluated the performance based on *F*_1_-scores as in the case of the prediction of mental health, the *F*_1_-score is a good reflection of how sparsity affects the models’ judgment in detecting positive and false cases. The models already shown in Table 4 remained, in addition to other models that have been mentioned in the literature to perform well on sparse data sets. Among the ML algorithms used in the literature, the best performance was shown by the RF, with an *F*_1_-score of 0.35, while the FLMS showed an *F*_1_-score 0.21 higher than that of the RF. Both MLP and AdaBoost performed close to the RF, with an *F*_1_-score of 0.33. The algorithm that performed the worst in handling sparsity was the SVM, with an *F*_1_-score of only 0.15. This experiment highlights the fact that due to the combination of modeling, the FLMS performs better when dealing with highly sparse small data sets (Table 7). The metadata of experiment 3 are shown in Table 8.

**Table 7:** Experiment 3 performance of the FLMS^a^ compared to common ML^b^ algorithms for tackling sparsity on the adolescent data set.

Predictive learning approach	Modeling strategy	Test *F*_1_-score, mean (SD)
FLMS	ATU^c^ + LOTPO^d^ + LOPO^e^ + LTXO^f^	0.56 (0.55)
XGBoost^g^ [14,17]	Leave 1 out	0.28 (0.57)
KNN^h^ [10,11,13,16]	Leave 1 out	0.30 (0.73)
MLP^i^ [9]	Leave 1 out	0.33 (0.70)
SVM^j^ [12]	Leave 1 out	0.15 (0.62)
DT^k^ [13]	Leave 1 out	0.24 (0.70)
RF^l^ [11,13]	Leave 1 out	0.35 (0.65)
AdaBoost^m^ [14]	Leave 1 out	0.33 (0.60)

^a^FLMS: framework for longitudinal multimodal sensors.

^b^ML: machine learning.

^c^ATU: accumulated time unit.

^d^LOTPO: leave one time unit one participant out.

^e^LOPO: leave one participant out.

^f^LTXO: leave time unit X out.

^g^XGBoost: Extreme Gradient Boosting.

^h^KNN: K-nearest neighbor.

^i^MLP: multilayer perceptron.

^j^SVM: support vector machine.

^k^DT: decision tree.

^l^RF: random forest.

^m^AdaBoost: Adaptive Boosting.

**Table 8:** Experiment 3 metadata.

Metadata	Experiment 3
Feature preprocessing	KNN^a^ imputation, dropping highly corelated columns, sklearn StandardScaler
Modeling CV^b^ strategy	FLMS^c^, leave 1 out
ML^d^ algorithms used	import XGBoost^e^ as xgb from sklearn.svm import SVM^f^ sklearn.neural_network import MLPClassifier sklearn.neighbors import KNeighborsClassifier sklearn.tree import DecisionTreeClassifier sklearn.ensemble import RandomForestClassifier sklearn.ensemble import AdaBoostClassifier
Random state	42
Evaluation metrics	*F*_1_-score

^a^KNN: K-nearest neighbor.

^b^CV: cross-validation.

^c^FLMS: framework for longitudinal multimodal sensors.

^d^ML: machine learning.

^e^XGBoost: Extreme Gradient Boosting.

^f^SVM: support vector machine.

## Discussion

### Principal Findings

Solving the problem of limited and sparse data sets is not a singular modeling-based endeavor. It requires flexibility and a combination of strategies to achieve predictions that can be trusted. In this section, we discuss our ranking framework’s overarching aims, performance, and limitations based on our assessments.

In experiment 1, we tested the FLMS in comparison to baseline user-agnostic and personalized models. Our framework achieved a higher accuracy, recall, and *F*_1_-score for the predictions when compared to singular modeling approaches, as seen in Table 2. We also demonstrated how we arrived at the sensor combination for the best set of predictions using hamming distances in stage 3 of the FLMS, as reflected in Table 3. In experiment 2, we compared the FLMS with SOTA algorithms used in the literature for predicting mental health states using sensors. The results from this experiment showed the FLMS to perform better than the existing algorithms in terms of accuracy, recall, and *F*_1_-scores (Table 4). Experiment 2 also highlighted the FLMS’s ability to reduce overfitting in comparison to the SOTA algorithms. The FLMS showed that the training accuracy and test accuracy did not diverge by large margins, indicating it had not been overfitting the models. Lastly, we compared the FLMS ranking with that of existing ML algorithms that perform well with sparse data in experiment 3. We saw that the data set we used in our experiments exhibited 35% sparsity, which is a significant amount in an already small data set. The FLMS had a higher *F*_1_-score compared to the rest of the ML algorithms.

### Comparison With Previous Research

The results of baseline modeling are consistent with previous studies [10,29] that showed superior performance when models were personalized. The increase in accuracy shows that our framework was able to narrow down the best set of predictions overall.

Hamming distance results showed that in LOPO and LTXO approaches, single-sensor deployment and a dual-sensor combination perform equally well as 6-sensor combinations and achieve a minimum hamming distance. This brings forth the advantage of our framework to prioritize sensor selection for yielding best predictions overall and for only the necessary number of feature sets.

The results of experiment 2 provide us with further evidence of the ranking frameworks’ efficacy in balancing reliance between both user-agnostic and personalized approaches. Despite a higher accuracy, the recall of the FLMS does not overfit like that of other SOTA ML algorithms. The FLMS uses weights to balance out such effects, thus reducing the impact of overfitting in prediction performance. The test with popular existing ML algorithms showed that, despite the success of the models in previous studies [9-11,13-17], they struggle when the data set is small and noisy, as is the case of the depression data set presented in this work. This performance result is similar when we look at the capability of ML algorithms that are better at handling sparsity. We found the FLMS to perform better than those algorithms.

Overall, seeking a single user-agnostic model that fits all is an elusive problem as most existing works suggest better performance for specialized approaches. However, specialized modeling does not perform well on heterogeneous data sets. Therefore, neither user-agnostic nor personalized modeling alone can be applicable to a specific problem area. Our framework provides a practical way to balance the 2 approaches, particularly for dealing with limited data sets.

### Limitations and Future Directions

We encountered a few limitations with this study that can be addressed in future work. The FLMS was tested on the case of depression in adolescents. As such, we have not been able to establish a lower bound on the data set size that our framework is capable of handling.

Another area that we could not elaborate on is the computing speed of such a framework that might be impacted if sensor numbers rise to higher levels. Lastly, the framework was equipped with lightweight and widely used ML algorithms. Methods such as the generalized linear mixed model (GLMM) for handling longitudinal data could not be tested.

Future work can address these limitations with exposure of the framework to more multimodal, longitudinal data sets and adapting and testing other ML algorithms. Interesting future directions for the framework include its online adaptation and a similarity-based cold-start solution.

### Conclusion

In this study, we presented a novel prediction-ranking framework for modeling limited noisy or sparse, multimodal, longitudinal passive sensor data. We tested our framework on an adolescent depression data set consisting of 45 participants over a period of 24 weeks. The results showed that despite the complexity and limitations of the data set, our framework is able to provide better predictions compared to singular modeling approaches. In experiment 1, our model achieved a 7% increase in accuracy and a 13% increase in recall. In experiment 2 with synthetic data, our model achieved a 5% increase in accuracy and avoided overestimating the recall value through ensembling predictions. The framework also showed its ability to explore sensor combinations through feature fusion. Our tests with existing popular SOTA algorithms showed that the models struggle when data tend to be limited and noisy. We also tested the FLMS with algorithms that perform well with sparsity and found the FLMS to exhibit a better performance. In conclusion, the FLMS can be an effective tool for passive sensing studies.

## Appendix

Multimedia Appendix 1 FLMS ranking overview. Algo: algorithm; ATU: accumulated time unit; avg: average; CV: cross-validation; FLMS: framework for longitudinal multimodal sensors; LOPO: leave one participant out; LOTPO: leave one time unit of participant out; LTXO: leave time unit X out; ML: machine learning.

## Abbreviations

AdaBoost: Adaptive Boosting
API: application programming interface
ATU: accumulated time unit
CV: cross-validation
DL: deep learning
DT: decision tree
FLMS: framework for longitudinal multimodal sensors
HAR: human activity recognition
KNN: K-nearest neighbor
LDA: linear discriminant analysis
LOPO: leave one participant out
LOTPO: leave one time unit one participant out
LR: linear regression
LSTM: long short-term memory
LTXO: leave time unit X out
ML: machine learning
MLP: multilayer perceptron
PCA: principal component analysis
PHQ-9: 9-item Patient Health Questionnaire
RF: random forest
SOTA: state of the art
SVM: support vector machine
XGBoost: Extreme Gradient Boosting
