# Supplementary figures and images for: Framework for Ranking Machine Learning Predictions of Limited, Multimodal, and Longitudinal Behavioral Passive Sensing Data: Combining User-Agnostic and Personalized Modeling
Source: JMIR AI. 2024 May 20;3:e47805. doi: 10.2196/47805 (PMC11148522; doi:10.2196/47805)

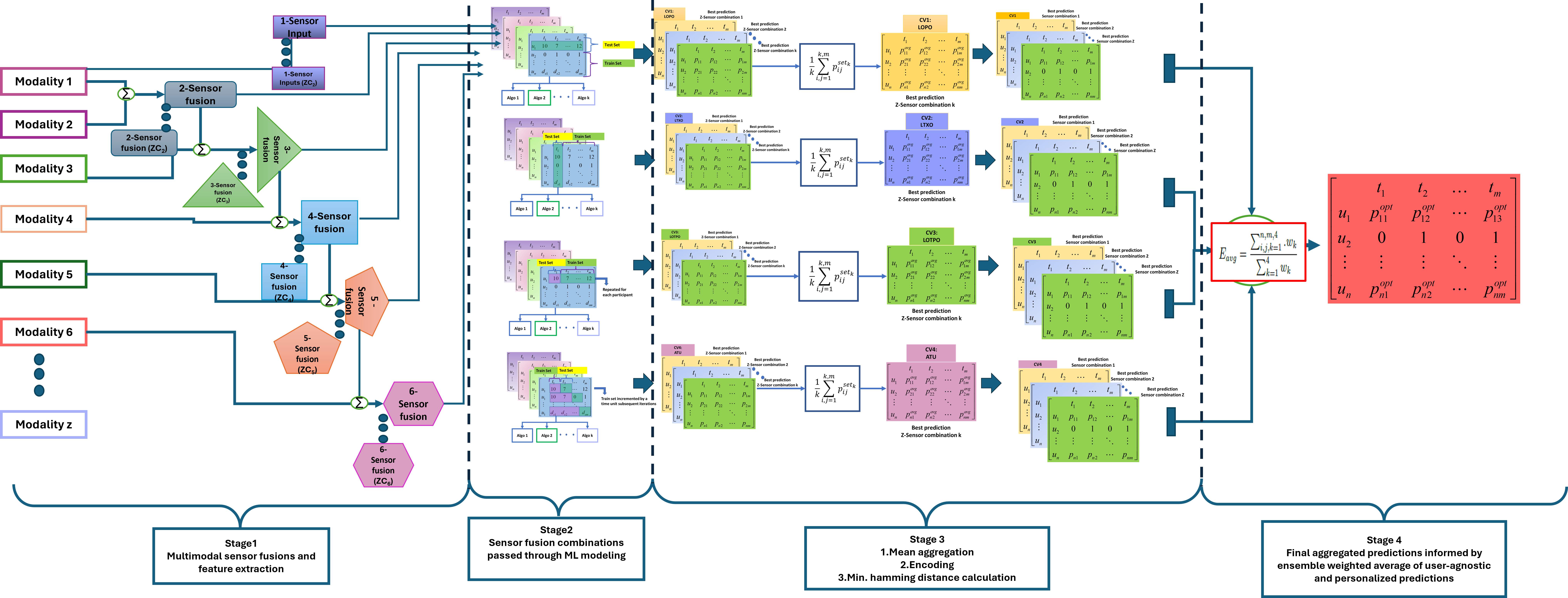

Supplement: Multimedia Appendix 1 [file ai_v3i1e47805_app1.png]
